# Gone with the heat: a fundamental constraint on the imaging of dust and molecular gas in the early Universe

**DOI:** 10.1098/rsos.160025

**Published:** 2016-06-08

**Authors:** Zhi-Yu Zhang, Padelis P. Papadopoulos, R. J. Ivison, Maud Galametz, M. W. L. Smith, Emmanuel M. Xilouris

**Affiliations:** 1 Institute for Astronomy, University of Edinburgh, Royal Observatory, Blackford Hill, Edinburgh EH9 3HJ, UK; 2 ESO, Karl-Schwarzschild-Strasse 2, 85748 Garching, Germany; 3 School of Physics and Astronomy, Cardiff University, Queen’s Buildings, The Parade, Cardiff CF24 3AA, UK; 4 Research Center for Astronomy, Academy of Athens, Soranou Efesiou 4, 115 27 Athens, Greece; 5 Institute for Astronomy, National Observatory of Athens, 15236 Penteli, Greece

**Keywords:** galaxies: interstellar medium, cosmology: cosmic background radiation, galaxies: high-redshift

## Abstract

Images of dust continuum and carbon monoxide (CO) line emission are powerful tools for deducing structural characteristics of galaxies, such as disc sizes, H_2_ gas velocity fields and enclosed H_2_ and dynamical masses. We report on a fundamental constraint set by the cosmic microwave background (CMB) on the observed structural and dynamical characteristics of galaxies, as deduced from dust continuum and CO-line imaging at high redshifts. As the CMB temperature rises in the distant Universe, the ensuing thermal equilibrium between the CMB and the cold dust and H_2_ gas progressively erases all spatial and spectral contrasts between their brightness distributions and the CMB. For high-redshift galaxies, this strongly biases the recoverable H_2_ gas and dust mass distributions, scale lengths, gas velocity fields and dynamical mass estimates. This limitation is unique to millimetre/submillimetre wavelengths and unlike its known effect on the global dust continuum and molecular line emission of galaxies, it cannot be addressed simply. We nevertheless identify a unique signature of CMB-affected continuum brightness distributions, namely an increasing rather than diminishing contrast between such brightness distributions and the CMB when the cold dust in distant galaxies is imaged at frequencies beyond the Raleigh–Jeans limit. For the molecular gas tracers, the same effect makes the atomic carbon lines maintain a larger contrast than the CO lines against the CMB.

## 1. Introduction

Imaging the thermal continuum from cosmic dust and the carbon monoxide (CO) line emission from its concomitant molecular (H_2_) gas at millimetre and submillimetre wavelengths has become a powerful tool for deducing structural characteristics of galaxies [1–7]. Moreover, the H_2_ gas velocity field together with its relative mass distribution in galactic discs are indispensable for studying instabilities in the evolution of such systems from the early Universe to the present day [5,6,8].

As the cosmic microwave background (CMB) temperature rises in the distant Universe, the ensuing thermodynamic interaction between the CMB and the cold interstellar medium (ISM; i.e. dust and H_2_ gas) becomes important and must be taken into account [9–11]. This effect is prominent at millimetre and submillimetre wavelengths, close to the blackbody peak of the CMB. The latter now becomes the dominant thermal pool with which the cold dust and H_2_ gas of distant galaxies interacts, while it also provides the irreducible background against which dust continuum and molecular line emission of gas-rich discs must be measured.

The effect of the CMB on the global (i.e. spatially integrated) spectral energy distributions (SEDs) of the dust emission and on the spectral line energy distributions (SLEDs) of CO line emission in high-redshift galaxies has been widely studied [9–11]. Its impact on measuring the total IR luminosity (∝ star-formation rate, SFR) for dust-enshrouded objects in the distant Universe and the total mass of dust and H_2_ is also well characterized [11]. However, an elevated CMB will also progressively erase the contrasts between the brightness distributions of dust continuum and molecular line emission emanating from the cold ISM of distant galaxies. This effect, and its impact on the recovered morphologies and dynamics of gas-rich discs in the distant universe have yet to be studied.

Large masses of cold dust and H_2_ gas (*T*_dust, kin_ approx. 15–25 K) are common in local galaxies [2,12–14] including the Milky Way [15]. In spirals, their distributions contain the bulk of the H_2_ gas and dust, define the total size of their H_2_ gas and dust discs and encompass all major star-forming (SF) activity [3,14]. The brightness distribution of cold dust often extends well past the CO-marked H_2_ gas disc [2,16], whereas the low-*J* CO line emission from cold H_2_ gas remains the best—i.e. the most mass-inclusive—probe of galactic dynamics in metal-rich discs after the Hi 21 cm line. There is no evidence that this picture changes much for distant gas-/dust-rich SF galaxies, where extended cold H_2_ gas reservoirs are now well established [17]. This is expected because, despite the fact that a large fraction of H_2_ gas and dust mass is involved in star formation in high-redshift galaxies (and will belong to a warm ISM phase), star formation remains a globally inefficient process in discs [5,18], leaving massive distributions of H_2_ gas and dust in a cold low-density state [19,20].

Determining scale lengths of gas discs [21], velocity fields of molecular gas [22], enclosed dynamical gas masses (*M*_dyn_) [19,23], Tully–Fischer relations, and the cosmic evolution of gas-rich disc instabilities [23,24] in high-redshift galaxies depends critically upon recovering the dust and H_2_ gas distributions, *irrespective of their thermal state*, i.e. warm SF versus cold non-SF gas and dust. The constraint set by the elevated CMB on the recoverable distributions of cold H_2_ gas and dust at high redshifts then leads directly to serious biases for some of the most important characteristics that are to be determined in exquisite details with Atacama Large Millimetre Array (ALMA) and the Karl G. Jansky Very Large Array (JVLA) [25,26]. The wavelength range covered by these facilities is where the elevated CMB has its strongest impact, namely centimetre, millimetre and submillimetre wavelengths, placing constraints on the morphological, structural and gas velocity field information that can be recovered by interferometer arrays.

The structure of this paper is as follows. In §2, we present the fundamental physics of the CMB effects on the dust continuum distributions. In §3, we simulate these CMB effects for the dust emission of three nearby galaxies over different redshifts, and describe the impact on their recoverable dust continuum brightness distribution. In §4, we study the effects of the CMB on the emission of molecular gas tracers, namely CO and C i transitions, and describe how the CMB effects change their observed brightness distributions and recoverable gas velocity fields for galaxies at high redshift. Finally, in §5, we give a summary and some concluding remarks.

## 2. Effects of the cosmic microwave background on the observed dust continuum brightness distributions

We consider the effects of the CMB on the dust continuum emission at different redshifts. Following [11], we keep the same interstellar radiation field (ISRF), the same intrinsic dust properties (i.e. column density, dust emissivity spectral index *β*). The only variable is the CMB temperature that increases with redshift as *T*_CMB_(*z*)=*T*_CMB_(0)×(1+*z*). We assume that the dust optical depth *τ*≪1 at radio/(sub)millimetre wavelengths. When the dust, the ISRF and the CMB reach a thermal equilibrium, the dust emission can be described with a modified blackbody (MBB)

2.1 $$\int_{0}^{\infty }\nu^{\beta }B_{\nu }(T_{d})\;d\nu \propto T_{d}^{4+\beta }.$$

The modelling includes two major effects—the CMB dust heating and the CMB background continuum subtraction. At a given redshift, dust is heated by the CMB photons to a temperature *T*_d_(*z*):

2.2 $$T_{d}(z)=T_{d}(0){\left\{1+[{\left(1+z\right)}^{4+\beta }-1]{\left[\frac{T_{\mathrm{CMB}}(0)}{T_{d}(0)}\right]}^{4+\beta }\right\}}^{1/(4+\beta )}.$$

In observed data, the background emission is always removed, by one or more kinds of background subtraction in single-dish observations or by Fourier spatial filtering in interferometric observations. The radiation temperature at the rest frequency, *ν*=*ν*_obs_(1+*z*), in *the source local rest frame* is *J*[*T*_d_(*z*),*ν*]−*J*[*T*_CMB_(*z*),*ν*], where $J(T,\nu )=(h\nu /k_{B})/[\exp(h\nu /k_{B}T)-1]$ is the Planck radiation temperature at a frequency *ν* and for a dust temperature, *T*_d_(*z*).

For a given galaxy at redshift, *z*, the brightness in the rest frame *B*_*ν*_∝(*J*[*T*_d_(*z*),*ν*]−*J*[*T*_CMB_(*z*),*ν*]), so the brightness ratio (at the same emitting frequency) between a galaxy at redshift *z* and the same galaxy at redshift 0 is

2.3 $$R_{B}=\frac{J[T_{d}(z),\nu ]-J[T_{\mathrm{CMB}}(z),\nu ]}{J[T_{d}^{z=0},\nu ]-J[T_{\mathrm{CMB}}^{z=0},\nu ]}.$$

**Figure 1.**
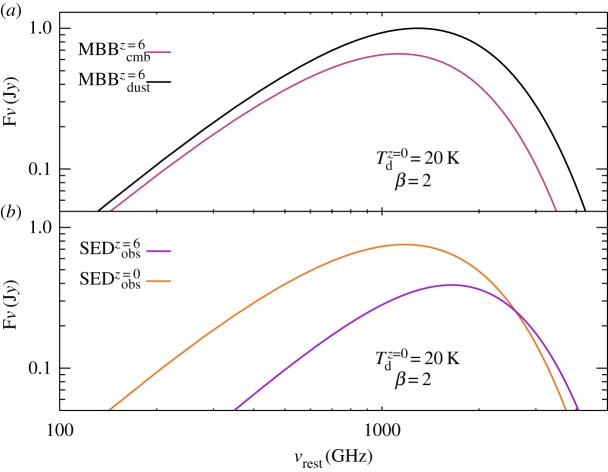
Spectral energy distributions (SEDs) of dust with *T*^*z*=0^_d_=20 *K*, *β*=2 and at *z*=6. Panel (*a*) shows the predicted modified black bodies (MBBs) of the CMB emission (red) and the intrinsic dust emission (black). Panel (*b*) shows the dust SEDs at *z*=0 (brown) and *z*=6 (purple), both after the background subtraction. All plots are normalized to the peak of the intrinsic dust emission at *z*=6.

In figure 1, we investigate the effects of the CMB on dust emission by comparing SEDs with the CMB at *z*=0 and *z*=6. We adopt a local galaxy with a ‘normal’ cold dust temperature of *T*^*z*=0^_d_=20 *K* and *β*=2, shift it to redshift *z*=6, and keep all parameters the same except for the CMB temperature. Figure 1*a* shows the SEDs of the CMB (red) and the intrinsic dust MBB (black) at redshift, *z*=6, and figure 1*b* shows the observed rest-frame dust SEDs (after background subtraction) at *z*=0 (brown) and *z*=6 (purple). At low frequencies (the Rayleigh–Jeans domain), as shown in figure 1*b*, the dust emission brightness at *z*=6 is lower than that at redshift 0, with the brightness difference *δI*_*ν*_/*I*_*ν*_∝*δT*/*T*, so that the near-identical temperatures of the dust and CMB blackbodies would (linearly) translate to near-identical brightness. The resulting brightness dimming effect will make it difficult to image cold dust emission distributions at high redshifts at low frequencies.

However, we find that beyond a certain frequency the observed contrast against the source-frame CMB increases again. This is because as the CMB heats up the dust to higher temperatures and brings their two SEDs ever closer, the rest-frame frequency crosses over to the Wien side of the two nearly identical SEDs. Then, the resulting brightness difference will be nonlinearly boosted with respect to the underlying small temperature difference between the two SEDs (*δI*_*ν*_/*I*_*ν*_>*δT*/*T*), over-compensating for the dimming owing to the ever-closer blackbody functions of the cold dust and the CMB (figure 1*a*).

An equivalent view of this effect using a galaxy at a given high redshift (rather than a galaxy ‘moving’ out to progressively higher redshifts) can be recovered if we consider ever higher-frequency observations of its cold dust continuum brightness distribution. Then, as long as the imaging observations are performed at a frequency high enough to have the source-frame frequency cross from the Rayleigh–Jeans to the Wien side of the cold dust distribution, the observed dust continuum distribution will re-brighten. *This effect can then serve as a clear indicator, showing that a low-brightness dust continuum distribution observed at low frequencies in a distant galaxy is due to the CMB bias rather than owing to low dust mass surface densities*. This re-brightening effect will typically occur when we observe a high-redshift cold dust disc at relatively high observing frequencies—from the high end of the submillimetre to the THz regime, and it could even permit the recovery of its cold dust mass distribution, which may be impossible for frequencies in the Rayleigh–Jeans domain.

**Figure 2.**
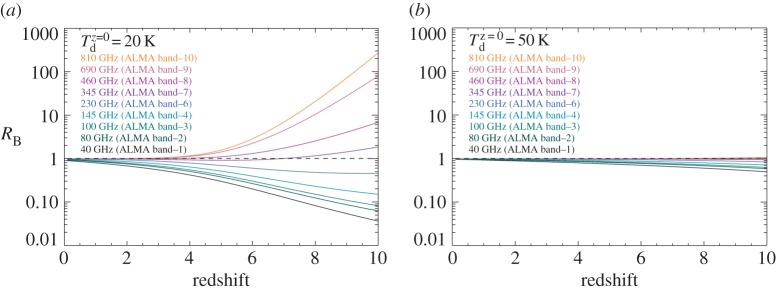
Predicted brightness ratios (*R*_B_; equation (2.3)) between the dust emission with the CMB at redshift, *z*, and with that at redshift 0, observed in different ALMA bands (*ν*_obs_=40, 80, 100, 145, 230, 345, 460, 690 and 810 GHz). The CMB effects include both the additional CMB heating on the dust and the background continuum subtraction. (*a*) The ratios with an assumption of *T*^*z*=0^_d_=20 *K*. (*b*) The ratios with an assumption of *T*^*z*=0^_d_=50 *K*.

Figure 2 presents the effects of the CMB on the dust continuum emission by comparing the emergent flux density at *z*=0 and that of various redshifts. We plot *R*_B_ (equation (2.3)) as a function of redshift for different ALMA bands (*ν*_obs_=40, 80, 100, 145, 230, 345, 460, 690 and 810 GHz). We adopt two intrinsic dust temperatures: $T_{d}^{z=0}=20\;K$ for the quiescent cold dust on the disc and *T*^*z*=0^_d_=50 *K* for the warm dust heated by star formation or active galactic nuclei. We find that *R*_B_ decreases with redshift at low frequencies (dimming) and increases with redshift at high frequencies (re-brightening). Both effects are much less pronounced in the high-temperature case (*T*^*z*=0^_d_=50 *K*). For *T*^*z*=0^_d_=20 *K* at *z*=8, the brightness in ALMA band 10 is an order of magnitude higher than that at *z*=0, whereas in ALMA band 3, the brightness deceases by a factor of ten compared with that at *z*=0. ALMA band 7 is the least affected observing frequency.

## 3. Effects of the cosmic microwave background on the observed brightness distributions of dust continuum in spiral galaxies

In this section, we use three spiral galaxies to demonstrate the impact of the elevated CMB on the brightness distribution of their dust continuum and thus on the recoverable morphology of their dust mass distributions. Rather than assuming a *T*_dust_ range—as was the past practice for such studies [9,11]—we will demonstrate the CMB effect on the observable brightness distribution of cold dust continuum at high redshifts by using real *T*_dust_ maps of galaxies in the local Universe. Such maps can now be obtained from the wealth of IR/submillimetre imaging data available from the *Spitzer Space Telescope* and the *Herschel Space Observatory*. We exploit NGC628, M33 and M31, for which there are high-quality *T*_dust_ maps over most of their extent [13,27,28] and whose levels of SF activity range from the vigorous, in NGC628 and M33 (*Σ*_SFR_ approx. 10^−3^ to 10^−2^ $M_{⊙}\;{\mathrm{yr}}^{-1}\;{\mathrm{kpc}}^{-2}$ [29,30]), to the quiescent M31 (*Σ*_SFR_ approx. 10^−4^ to 10^−3^ $M_{⊙}\;{\mathrm{yr}}^{-1}\;{\mathrm{kpc}}^{-2}$ [31]).

### (a) Archival *Herschel* data

To model the cold dust emission in high-redshift galaxies, we exploit 500 μm images from the *Herschel* science archive system. The galaxies were observed using the Spectral and Photometric Imaging Receiver (SPIRE) [32]. For NGC 628, the data were obtained as a part of the KINGFISH project^1^ (P.I. Kennicutt; observation ID 1342179050). The M 33 data were observed in the HerM33es project^2^ (P.I. Kramer; observation ID 1342189079). The M 31 data were observed in the HELGA project^3^ (P.I. Fritz; observation ID 1342211294).

### (b) Temperature maps of cold dust distribution

Dust temperatures are often derived from fitting an MBB. Most galaxies, however, contain a range of dust temperatures. The warm dust component (*T*_d_ approx. 40–100 K) often contains less mass, but contributes significantly to the fitting of single MBB, leading to an overestimate of the cold dust temperature. Dual-MBB model fits can better constrain the temperature for the cold dust component, which is where the effect of the CMB plays an important role [27]. We simplify the fitting procedures in the literature [13,27,28] and repeat the fit to derive cold dust temperature maps for M31, M33 and NGC 628, by fixing the emissivity indices for both cold and warm components. Here we do not consider any variation of *β* within the galaxies, to minimize the degeneracy between the dust temperature and *β*. The adopted value, *β*=2, is often used for modelling high-redshift galaxies globally. This assumption allows us to see how the dust temperature is changed solely by the CMB effect. We obtain a robust determination of the cold dust temperature distribution based on maps at multiple wavelengths. We exclude pixels below the 3*σ* detection limit in all bands.

From the *T*^*z*=0^_dust_ maps, we obtain *T*^*z*^_dust_ maps at a given redshift *z*, accounting for the CMB dust heating [9–11]. In order to show solely the effect of the CMB on the emergent dust continuum brightness distribution in the source rest frame we do not apply the cosmological correction *I*_*ν*_obs_(*r*)_=(*ν*_obs_/*ν*_em_)^3^ *I*_*ν*_em__(*r*), where *I*_*ν*_em_(*r*)_ is the source rest-frame brightness distribution. Doing so would introduce another (1+*z*)^−3^ dimming that could make it even harder to discern the CMB-immersed brightness features. Finally, to obtain the brightness images at various observing frequencies, we scale the *Herschel* 500 μm images by the factor $R_{B}^{\prime }$ (equation (3.1)) at both redshift *z* and *z*=0:

3.1 $$R_{B}^{\prime }={\left(\frac{\nu }{\nu_{500\text{μ}m}}\right)}^{\beta +2}\frac{J[T_{d}(z),\nu ]-J[T_{\mathrm{CMB}}(z),\nu ]}{J[T_{d}^{z=0},\nu_{500\text{μ}m}]-J[T_{\mathrm{CMB}}^{z=0},\nu_{500\text{μ}m}]},$$

where $J(T,\nu )=h\nu /k_{B}/[\exp(h\nu /k_{B}T)-1]$ is the Planck radiation temperature at frequency, *ν*, with a temperature, *T*. *β* is the dust emissivity index. We fix *β*=2. Varying *β* does not change our conclusions.

**Figure 3.**
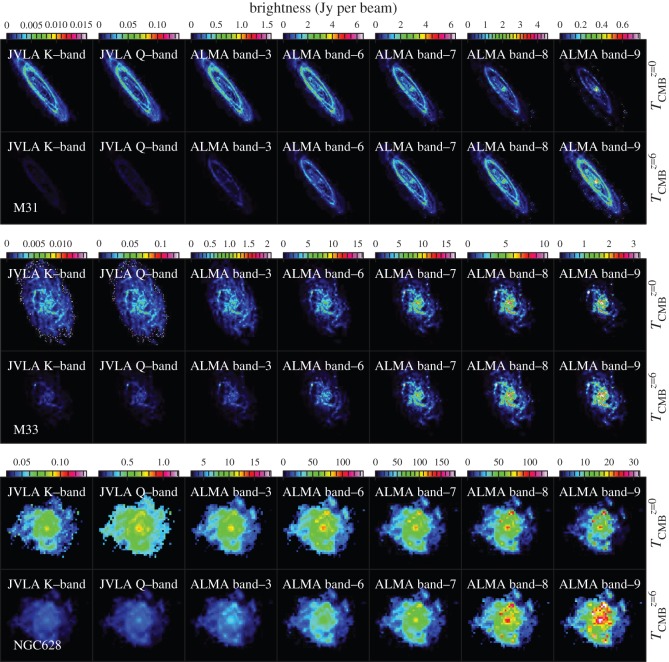
Simulated images of the cold dust continuum emission of M31, M33 and NGC628 at *z*=6, observed in different bands (25, 45, 100, 230, 345, 460, 690 GHz) of the JVLA and ALMA. The images are scaled from 500 μm *Herschel* images at *z*=0 with the $R_{B}^{\prime }$ factors, and are displayed in the source rest frame. For each galaxy, we plot the continuum emission with the CMB temperatures of *z*=0 ($T_{\mathrm{CMB}}^{z=0}=2.725\;K$; top panels) and *z*=6 ($T_{\mathrm{CMB}}^{z=6}=19.075\;K$; bottom panels).

We compare the re-scaled maps for the CMB at *z*=0 and *z*=6 as an example. The re-scaled images are shown in figure 3 and they correspond to the dust emission brightness distributions at the rest frequency *ν* *in the source rest frame*, i.e. what an observer at the given redshift would see. This isolates the effect of the enhanced CMB on the observed brightness distribution from all the other factors that also affect it, e.g. cosmological size changes, (1+*z*)^−3^ dimming, telescope sensitivity, etc. We then examine the relative changes before and after considering the CMB effects in these galaxies. This simple re-scaling assumes that (i) the dust emission is optically thin and (ii) dust optical depths do not have any dependence on $T_{\mathrm{dust}}^{z}$. These hold for the dust emission in local galactic discs.

From figure 3, the CMB effect on the brightness distributions of the observed dust emission is obvious, with the contrast between the cold dust emission and the CMB gradually disappearing at relatively lower frequencies. Besides the ‘shrinking’ of the observed sizes of the dust emission in all three galaxies (expected because the cold dust phase normally traces the most extended regions of galactic discs), we also see secondary effects where the warm/cold dust emission contrast changes *within* the observed discs. We note that for ALMA band 7 observations the global scale lengths and the dust structures are only modestly affected by the CMB, because it lies in the transition zone between the Rayleigh–Jeans and the Wien regimes for the cold dust of these galaxies (see §2).

**Figure 4.**
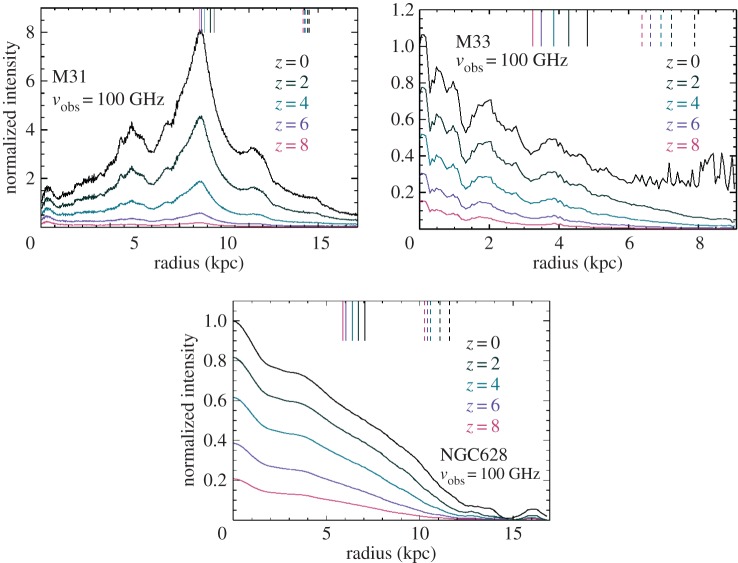
Radial distributions of the dust continuum emission of M31, M33 and NGC628 emitting at an observing frequency of 100 GHz at redshifts 0, 2, 4, 6 and 8, respectively, shown in different colours. We make concentric elliptical rings with the inclination and position angles of these galaxies, and obtain the average flux in each ring. The radial distribution plots are then normalized to emission at the central positions of the galaxies at *z*=0. The vertical solid lines show the half-light radii (*R*_50_), and the vertical dashed lines show the 90% light radii (*R*_90_).

In figure 4, we plot the radial distributions of the dust continuum emission at the observing frequency of 100 GHz at *z*=0, 2, 4, 6 and 8, respectively. The colours show the redshifts. We make concentric elliptical rings with the inclination and position angles of these galaxies, and obtain the average flux within each ring. The radial distribution plots are then normalized to the flux in the central positions of galaxies at *z*=0. The vertical solid lines show the half-light radius (*R*_50_), within which half of the total flux of the galaxy is found. The vertical dashed lines show the 90% light radius (*R*_90_).

The effect of the CMB on the observed spatial distributions of dust masses is important, because such morphological characteristics are often used to infer whether disc instabilities are driving star formation in discs. In the case of the globally cold M31, the effect of a rising CMB is most dramatic, with the entire galaxy ‘fading’ into the CMB. It is worthwhile to point out that the *R*_50_ and *R*_90_ radii are measured without taking noise into account, so they only show the relative change between the central region and disc when we have unlimited sensitivity. The effect of the CMB on the observed dust continuum distributions and scale lengths is obvious. Here, we must also note that the cold dust component will be present whenever dust lies far from radiation sources (stars or active galactic nuclei) that could warm it, like the dust reservoirs expected in the gas outflows or inflows found in the local and the distant Universe [33–35]. Thus, the effect of the rising CMB on the brightness distributions of dust emission will not be confined solely to galactic discs.

## 4. Effects of the cosmic microwave background on the observed morphology and kinematics of the molecular gas

In what follows, we investigate the impact of the CMB on molecular gas mass tracers, namely the CO and C i emission lines, and on the H_2_ gas velocity fields marked by CO and C i. We select the NGC628 galaxy, for which a large and fully sampled *J*=2–1 map is available [36]. We consider the lowest *J* transitions of CO, i.e. the *J*=1–0, 2–1 and 3–2 lines. The first two trace the global H_2_ gas in galaxies, irrespective of the thermal state or density (as long as $n≳10^{3}\;{\mathrm{cm}}^{-3}$, which is the case for most CO-rich H_2_ gas in galaxies), and are thus the spectral lines of choice when unbiased views of H_2_ gas mass and H_2_ velocity fields are sought. CO *J*=3–2 is the last CO transition to have non-negligible contributions from the cold, low-density, non-SF gas, though most of its luminosity comes from the warm and dense H_2_ gas found near SF sites. Any higher-*J* transitions are unsuitable as global tracers of H_2_ gas mass and velocity fields. We also compute the CMB effects on the two neutral atomic carbon lines, C i 1–0 and 2–1, which have been shown to be also good tracers of H_2_ gas mass and galactic dynamic mass, potentially better than the low-*J* CO transitions [37–39]. Cii is an excellent tracer of gas dynamics and is barely affected by the CMB; however, the Cii emission mostly arises from both photon-dominated regions and Hii regions, making it not solely sensitive to the H_2_ gas [40].

We do not have a multiplicity of CO lines per position within NGC628 that would allow us to determine *n*_H_2__, *T*_kin_ and d*v*/d*r* (the average gas velocity gradient, which depends on the dynamical state of the H_2_ gas). These parameters, once determined, could allow the computation of the emergent CO line intensities under different CMB backgrounds using a typical large velocity gradient (LVG) radiative transfer approach. However, the lack of spatially resolved CO SLEDs necessitates a more conservative approach where we set: kinetic temperature *T*_kin_=*T*_d_, H_2_ number density *n*_H_2__=10^3^ cm^−3^ and velocity gradient d*v*/d*r*=1 km s^−1^ pc^−1^, which are typical for quiescent non-SF H_2_ clouds in local spiral galaxies. We use the available *T*^*z*^_d_ maps as input to a standard LVG code to compute the emergent CO line brightnesses scaled from the available local CO *J*=2–1 map. Unlike the case for dust emission and its optical depths, the optical depths of CO lines do depend on *T*^*z*^_kin_ and thus do not cancel out.

### (a) Archival carbon monoxide data

We adopt the existing fully sampled CO *J*=2–1 data cube of NGC628 in the HERA CO-line extragalactic survey (HERACLES) archive.^4^ The data reduction has been described in [36]. The data cube has been converted to the main beam temperature scale, which represents the brightness temperature of CO lines. The angular resolution is approximately 13′′, corresponding to a linear scale of approximately 0.6 kpc.

### (b) Temperature maps of carbon monoxide

In order to match the high angular resolution CO data of NGC628, we convolve all the data to an angular resolution of 18′′ (the resolution of the *Herschel* 250 μm image), and generate a *T*_d_ map without the 350 and 500 μm data. The new *T*_d_ map is similar to the previous low-resolution *T*_d_ map adopted in modelling the dust emission (including the 350 and 500 μm data; see §3), with less than 15% difference in *T*_d_.

### (c) Radiative transfer modelling with large velocity gradient

In order to model the excitation conditions of molecular gas, which may not be in a local thermal equilibrium, the radiative transfer should be solved simultaneously for multiple population levels. We adopt a commonly used LVG assumption [41] wherein the line emission affected via self-absorption or induced emission only occurs in a local region, and the size is of the order of the local velocity dispersion (thermal and micro-turbulent) divided by a velocity gradient, d*v*/d*r*.

We used the LVG code, myradex,^5^ to model the CO ladders and determine the predicted CO line intensities. We adopt the geometry of a uniform radially expanding sphere, which has an escape probability of [1−exp⁡(−τ)]/τ, where *τ* is the optical depth of a given transition. We use the molecular collisional rates from the Leiden atomic and molecular database (LAMDA).^6^ The relative abundances to H_2_ are 8×10^−5^ for CO and 5×10^−5^ for C i, respectively [42]. We adopt a H_2_ number density of 10^3^ cm^−3^, which is a representative value for the bulk of molecular gas. We further assume the molecular gas is in a virialized condition, and set d*v*/d*r* to be 1 km s^−1^, because typically quiescent non-SF H_2_ clouds in galaxies are self-gravitating. The CMB temperature, *T*^*z*^_CMB_, varies with redshift as $T_{\mathrm{CMB}}^{z}=T_{\mathrm{CMB}}^{z=0}\times (1+z)$, where *T*^*z*=0^_CMB_ is 2.73 K. Using these inputs at *z*=0, we obtain the *R*^line^_B_ factor at various temperatures and redshifts:

4.1 $$R_{B}^{\mathrm{line}}=\frac{J[T_{\mathrm{ex}}^{\mathrm{line}}(z),\nu^{\mathrm{line}}]-J[T_{\mathrm{CMB}}(z),\nu^{\mathrm{line}}]}{J[T_{\mathrm{ex}}^{\mathrm{CO}J=2-1}(0),\nu^{\mathrm{CO}J=2-1}]-J[T_{\mathrm{CMB}}(0),\nu^{\mathrm{CO}\;J=2-1}]}\left[\frac{1-e^{-\tau_{\mathrm{line}}(z)}}{1-e^{-\tau_{\mathrm{CO}\;J=2-1}(0)}}\right].$$

**Figure 5.**
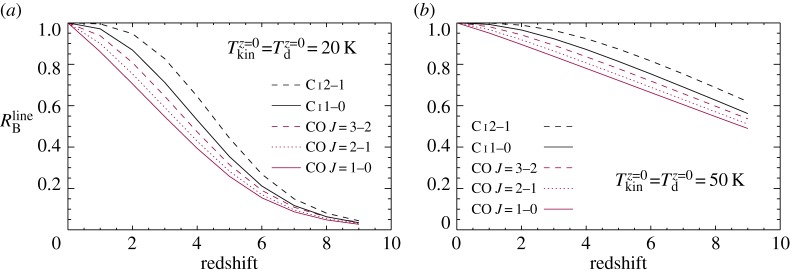
Predicted brightness ratios of line emission (*R*^line^_B_) between at redshift *z* and at *z*=0. The ratios are obtained using LVG modelling that assumes a molecular density of 10^3^ cm^−3^ and virialized conditions. (*a*) *R*^line^_B_ when assuming $T_{\mathrm{kin}}^{z=0}=T_{d}^{z=0}=20\;K$. (*b*) *R*^line^_B_ when assuming $T_{\mathrm{kin}}^{z=0}=T_{d}^{z=0}=50\;K$.

As in the case for the dust continuum emission, we have the (source)-(CMB) brightness terms, but the optical depth terms for the line emission now do not cancel out. In figure 5, we investigate the effects of the CMB on the H_2_ gas tracers by comparing their brightness at local and at various redshifts. We plot *R*^line^_B_ as a function of redshift for different transitions of CO and C i. Then, we apply equation (4.1) to the *z*=0 CO *J*=2–1 map in order to scale it to other CO transitions and to the two transitions of C i at different redshifts.

**Figure 6.**
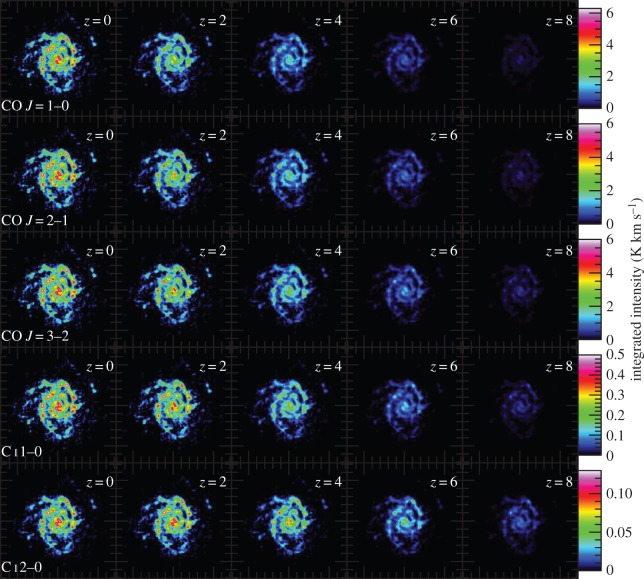
Simulated velocity-integrated brightness temperature (moment-0) maps of the CO and C i lines of NGC628, at different redshifts. The images are displayed in the source rest frame with units of K km s^−1^. From top to bottom: CO *J*=1–0, CO *J*=2–1, CO *J*=3–2, C i 1–0 and C i 2–1. We assume that *T*_kin_=*T*_d_ and that the H_2_ gas is virialized with a uniform number density of 10^3^ cm^−3^.

In figure 6, we show the velocity-integrated (over the entire velocity range) brightness temperature distribution of CO *J*=1–0, 2–1, 3–2 and C i 1–0, 2–1 lines in the source rest frame, for different redshifts. The effect of the CMB is again obvious, with the contrast of the CO lines from the cold molecular gas distributions diminishing for redshifts *z*≥2. We note that our conservative approach will artificially suppress the emergent CO *J*=1–0, 2–1, 3–2 line brightnesses from the warm/dense SF regions of NGC628. This is because *T*_kin_ is often significantly higher than *T*_dust_ for the H_2_ gas in SF regions, and d*v*/d*r* is also much larger than the quiescent value adopted. Thus, the actual contrast observed between the CO line brightness from the warm H_2_, and the CMB-affected cold H_2_ gas regions at high redshifts, will be even higher than that shown in figure 6. This then makes CMB-affected CO line brightness distributions even more biased, with our simulation presenting only the minimum effect. In a real galaxy with clumpy star formation regions, the underlying H_2_ mass distribution will be even harder to recover, with only the warm/dense ‘spots’ detected, whereas the colder, CMB-affected regions may be not detectable. *Such CMB-affected images of CO lines would imply an underlying H_2_ distribution clumpier than it actually is*.

On the other hand, figure 6 shows that the two C i lines do retain a higher brightness contrast against the CMB at high redshifts than the low-*J* CO lines. This happens for reasons similar to those behind the re-brightening of dust continuum distributions at high source-frame frequencies. Indeed, the upper level energies of the two C i lines correspond to *hν*_10_/*k*_B_∼24 K and *hν*_21_/*k*_B_∼62 K, which are $≳T_{\mathrm{kin}}(z)$ for cold molecular gas (especially for the C i 2–1 line). The relatively high frequencies of the C i lines make the line-CMB contrast significant as these two lines fall on the Wien side of the SLED expected for cold H_2_ gas (with the low-*J* CO lines being decisively on its Raleigh–Jeans domain). One may wonder why the high-*J* CO lines such as CO *J*=6–5, 7–6 with their $h\nu_{j\to j-1}/k_{B}$ factors also $≳T_{\mathrm{kin}}(z)$ for cold molecular gas (and thus on the Wien side of its expected CO SLED) could not be used in the same manner as the two C i lines. This is because, unlike the low critical densities of the two C i lines (*n*_crit_∼(300 to 10^3^) cm^−3^), those of high-*J* CO lines are ≳10^4^ cm^−3^. Thus, such CO lines will only be excited in the dense molecular gas, associated with the typically much more compact SF regions of galactic discs.

**Figure 7.**
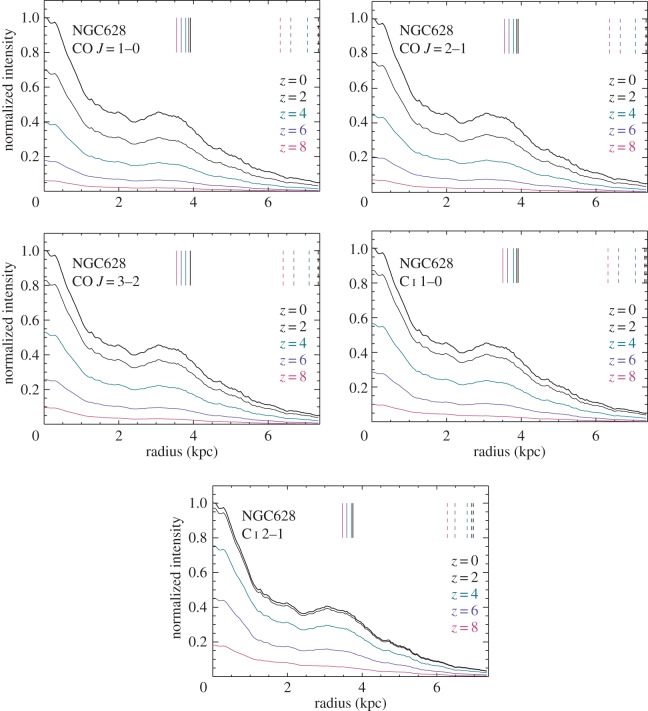
Radial distributions of the simulated CO *J*=1–0, CO *J*=2–1, CO *J*=3–2, C i 1–0 and C i 2–1 emission of NGC 628 at redshift 0, 2, 4, 6 and 8, respectively. The colours show the redshifts. Similar to figure 4, we measure the average flux in each concentric elliptical rings, and normalize it with the flux in the central position at *z*=0. The vertical solid lines show the half-light radii (*R*_50_), and the vertical dashed lines show the 90% light radii (*R*_90_).

In figure 7, we plot the radial distributions of the line emission of CO *J*=1–0, CO *J*=2–1, CO *J*=3–2, C i 1–0 and C i 2–1 in NGC628 at redshift of 0, 2, 4, 6 and 8, respectively. Using the images shown in figure 6, we measure the average flux density in concentric elliptical rings. We can see that the CMB effect is dramatic, diminishing the contrast of the cold gas line emission distribution against the CMB much more than that for the warm gas. As in the case of the cold dust, line imaging observations would then need much longer integration time to recover the CMB-dimmed cold molecular gas distributions in galaxies. On the other hand, they would readily recover the smaller distributions of warmer molecular gas, thus assigning potentially much smaller sizes to the gas discs of CMB-affected galaxies. *The latter would seriously underestimate their true underlying dynamical mass, and affect the deduced* *M*(*H_2_*)/*M*_dyn_ *ratio*.

**Figure 8.**
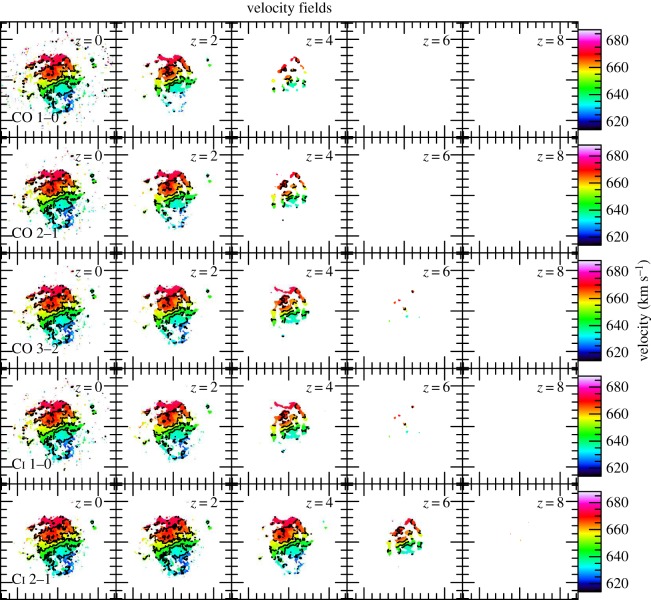
Simulated velocity field (moment-1) maps of CO *J*=1–0, CO *J*=2–1, CO *J*=3–2, C i 1–0 and C i 2–1 emission of NGC628 at different redshifts. The images are displayed in the source rest frame. The observing frequency varies with redshift and transition. The contours are from 630 to 690 km s^−1^ with steps of 10 km s^−1^. We use a cut-off of *I*_line_>3*σ* in generating all the velocity-field maps.

Finally, ALMA and JVLA promise not merely sensitive imaging of the dust and H_2_ gas mass distributions in distant gas-rich galaxies, but also the imaging of their H_2_ velocity fields. These, along with structural parameters such as gas-disc scale lengths and gas and stellar mass surface densities, are sensitive to disc instabilities in distant galaxies and yield critical information such as *M*_dyn_. Moreover, molecular gas velocity fields derived from CO lines are often used in order to decide the type of a distant heavily dust-obscured galaxy (disc versus merger). Interferometric line imaging observations designed to obtain high-quality H_2_ velocity field maps of distant galaxies are the most demanding kinds. This is because, unlike dust continuum or velocity-integrated line brightness images, a high signal-to-noise ratio must be achieved within narrow velocity channels in order to recover gas velocity field information. In figure 8, we show the effects of the CMB on such a set of mapping observations for CO *J*=1–0, 2–1, 3–2 and C i 1–0, 2–1. As the redshift increases, the recoverable map of the molecular gas velocity field shrinks revealing the most dramatic impact of the CMB bias on the recoverable information from molecular line imaging observations of distant galaxies. This in turn will strongly impact all galactic quantities derived from such gas velocity maps such as dynamical mass and the Toomre *Q* criterion used to decide the stability of gas discs.

Here we must note that once the noise associated with any synthesis imaging observations and the cosmological (1+*z*)^−3^ brightness dimming factor are taken into account, the fundamental constraints set by the rising CMB on the imaging of cold dust and molecular gas distributions of distant galaxies will take effect for redshifts lower than those indicated in this work. We will explore this issue using realistic simulations of ALMA/JVLA synthesis observations in a future paper.

## 5. Summary and conclusion

We uncover and describe a fundamental constraint placed by the CMB on centimetre/millimetre/submillimetre imaging observations of the cold dust and molecular gas distributions for galaxies in the distant Universe. The elevated CMB at high redshifts dramatically diminishes the emergent continuum and line brightness distributions of the cold gas and molecular gas. This in turn induces strong biases on the recoverable information such as the deduced molecular gas and dust disc distribution scale lengths, the CO-derived velocity fields, the enclosed dynamical mass estimate, the value of the Toomre *Q* parameter, and the observed dust and H_2_ gas mass distributions in galactic discs at high redshifts.

This constraint is unique to centimetre, millimetre and submillimetre wavelengths as the CMB at near-IR/optical wavelengths is negligible over the cosmic time during which H_2_/dust-rich galaxies are expected. Unlike the spatially integrated effects [9–11], this limitation placed by the CMB on the dust continuum and line brightness distributions cannot be addressed simply. Nevertheless, we find a unique signature that can identify CMB-affected dust continuum or line emission brightness distributions in high-redshift galaxies, and even recover some of the structural/dynamical information ‘erased’ by the elevated CMB. It consists of a nearly constant and then rising contrast between the dust or line brightness distribution and the CMB as the rest frame frequency of the imaging observations crosses over from the Raleigh–Jeans to the Wien domain of the cold dust and gas S(L)ED.

The fundamental constraint set by the CMB on the imaging of cold dust and molecular gas in the early Universe can also have a strong impact on the cosmological census of gas-rich galaxies. This is simply because galaxies that can be (cold-ISM)-dominated (e.g. isolated SF spirals) may be under-represented with respect to more (warm-ISM)-dominated galaxies (merger/starbursts) for surveys conducted in frequencies at the Raleigh–Jeans regime of the cold ISM emission S(L)ED.

The astronomical community was compelled to build ALMA by the natural desire to understand the how our home, the Milky Way, has evolved since the dawn of the cosmos. Indeed, the design of ALMA was driven to a significant extent by a requirement to trace the dynamics of gas in the precursors of relatively normal galaxies such as the Milky Way—seen in the days when the Solar System was forming, in a reasonable integration time, approximately 1 day. It is clear now that the dramatic effects of the CMB will need to be considered carefully when designing and conducting this experiment, and that studies of molecular gas and dust in the early Universe are more complicated than we had previously thought. The reliable detection of colossal cold, dusty structures [43]—lurking, unseen, having been driven out of massive galaxies by super-winds—was thought to be at the bleeding edge of what is currently possible, limited by the poor sensitivity of warm single-dish telescopes in space and interferometric studies that are ‘blind’ on scales above approximately 100 kpc. Now we understand that these technological issues may be the least of our concerns. To faithfully discern the bulk of the gas against the glow of the CMB, and in particular to reliably determine its dynamics, will require a considerable investment of time with the fully completed ALMA, with an emphasis on the gas tracers that we have shown to be relatively immune to CMB-induced biases, such as C i lines and continuum observations at a wavelength of about 1 mm.

## Footnotes

1 Key Insights on Nearby Galaxies (http://www.ast.cam.ac.uk/research/kingfish).

2 Herschel M33 Extended Survey (http://www.iram.es/IRAMES/hermesWiki/FrontPage).

3 The Herschel Exploitation of Local Galaxy Andromeda (http://www.astro.cardiff.ac.uk/research/astro/egalactic/surveys/?page=HELGA).

4 See http://www.mpia.de/HERACLES.

5 Myradex adopts the same equations as RADEX, and has no convergence problems, which appear in RADEX in certain parameter space: https://github.com/fjdu/myRadex.

6 See http://home.strw.leidenuniv.nl/moldata.

## Data accessibility

The work presented is based on archival data which are publicly available.
